# Supplementary material for: Drivers of Antarctic sea ice advance
Source: Nat Commun. 2023 Oct 5;14:6219. doi: 10.1038/s41467-023-41962-8 (PMC10556036; doi:10.1038/s41467-023-41962-8)
Supplement: Supplementary file 1 — Supplementary Information [file 41467_2023_41962_MOESM1_ESM.pdf]

Supplementary Information

Drivers of Antarctic sea ice advance

Kenza Himmich, Martin Vancoppenolle, Gurvan Madec, Jean-Baptiste Sallée, Paul R. Holland, Marion Lebrun

Supplementary Text

Analysis of the Supplementary Figures

The sea ice concentration budget at the date of advance (d_a) has large uncertainties, hence we integrate it in time over a time window spanning part of the sea ice season. In Supplementary Fig.1, we find that the sea ice concentration budget-derived limit between the inner and outer zones does not strongly depend on the size of the time integration window, suggesting it is robust enough for the present analysis.

In Supplementary Fig. 2, we evaluate the robustness of the sea surface temperature at date of advance (SST_{da}) across time periods and SST products. We find that SST_{da} slightly differs according to the averaging period (2003-2010 or 1982-2018) or the product (ESA CCI or Reynolds'). However, the effect of the inner-outer zone limit is always visible. Hence, the SST-based inner-outer zone limit is also robust, we reckon. Furthermore, SST_{da} - T_f (where T_f is the freezing temperature) is generally higher than uncertainty, and even more so in the outer zone. This is consistent with a higher SST_{da} in the outer zone compared to the inner zone. In the inner zone, higher than uncertainty (SST_{da} - T_f) could be due to a spatial averaging effect: a 15% ice covered grid point of 25 km^2 is not necessarily at the freezing point over its full surface.

In Supplementary Fig. 3, we test whether available in-situ hydrographic records support satellite-based findings regarding the SST at the date of advance. In-situ SST measurements are from float (2000-2020) and marine mammal-borne sensors (2004-2020, see Methods). In situ SSTs were collocated with the passive-microwave dates of sea ice advance for the corresponding sampling year. Only 28 records correspond to an available date of advance within 3 days in the corresponding satellite pixel. We find the histograms of Supplementary Fig. 2 from these 28 in situ records and their satellite counterparts (ESA CCI) SST_{da} to be compatible.

In Supplementary Fig. 4, we examine the temperature contribution to the stratification at the base of the mixed layer during the sea ice advance season (N_T^2). N_T^2 is proportional to the vertical temperature gradient at the base of the mixed layer and defined as: $N_T^2 = g\alpha\frac{\partial T_b}{\partial z}$, where T_b is the temperature at the base of the mixed layer, α, the thermal expansion coefficient at constant pressure: $\alpha = -\frac{1}{\rho}\frac{\partial \rho}{\partial t}$ and g, the gravity acceleration. $N_T^2 < 0$ ($N_T^2 > 0$) indicates a negative (positive) temperature gradient and an unstable (stable) temperature profile at the base of the mixed layer.

We find that, in the outer zone, the temperature profile at the base of the mixed layer is unstable during the three first months of the advance season ($N_T^2 < 0$), indicating a possible entrainment of warm waters into the mixed layer during these months. This entrainment might contribute to the excess of heat causing the high SST_{da} and the resulting melting in the outer zone.

In Supplementary Fig. 5, we show a strong correspondence between the seasonal maximum of mixed layer heat content (MLH_{max}) and the net radiative flux absorbed by the ocean. The latter is itself constrained by the date of sea ice retreat, which suggests a large control of MLH_{max} by radiative heating of the upper ocean and explains why MLH_{max} is tightly linked to the date of sea ice retreat (d_r) (see Figure 3c).

In Supplementary Fig. 6, we test the strength of the d_r-MLH_{max}-d_a linear relationships in the outer zone. We find that these relationships are significantly weaker in the outer zone than in the inner zone (see Fig. 3), according to Fisher's Z-test at a 0.01 significance level. However, the MLH_{max}-d_a relation still explains a large part (83%) of the variance in the climatological date of advance. By contrast, the d_r-d_a relation is weaker than the d_r-MLH_{max} and the MLH_{max}-d_a relations, in the outer zone.

In Supplementary Fig. 7, we show that the weaker d_r-d_a relation in the outer zone might be due to different spatial distributions of the errors in the d_r-MLH_{max} and the MLH_{max}-d_a linear regressions.

Supplementary Figures

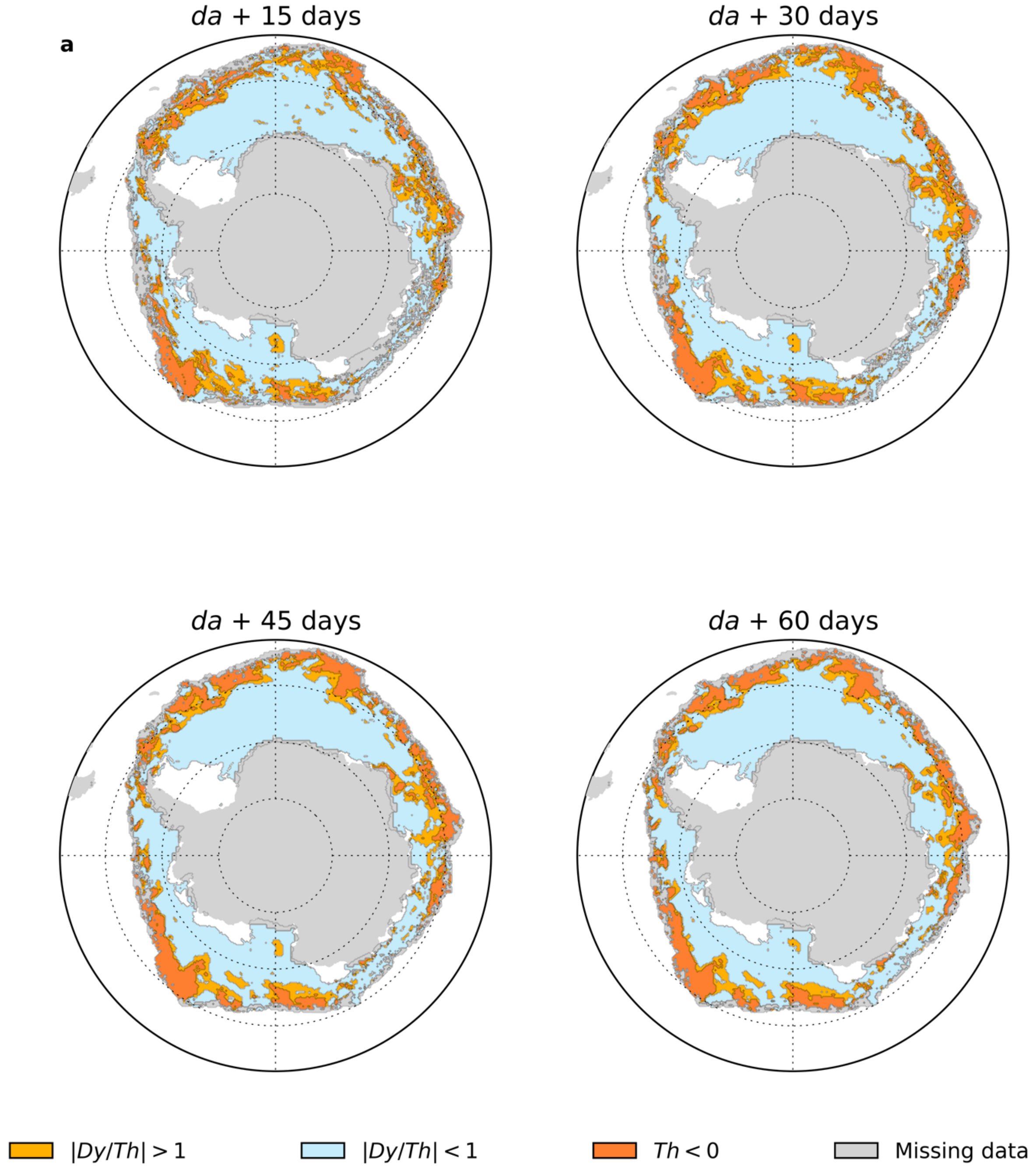

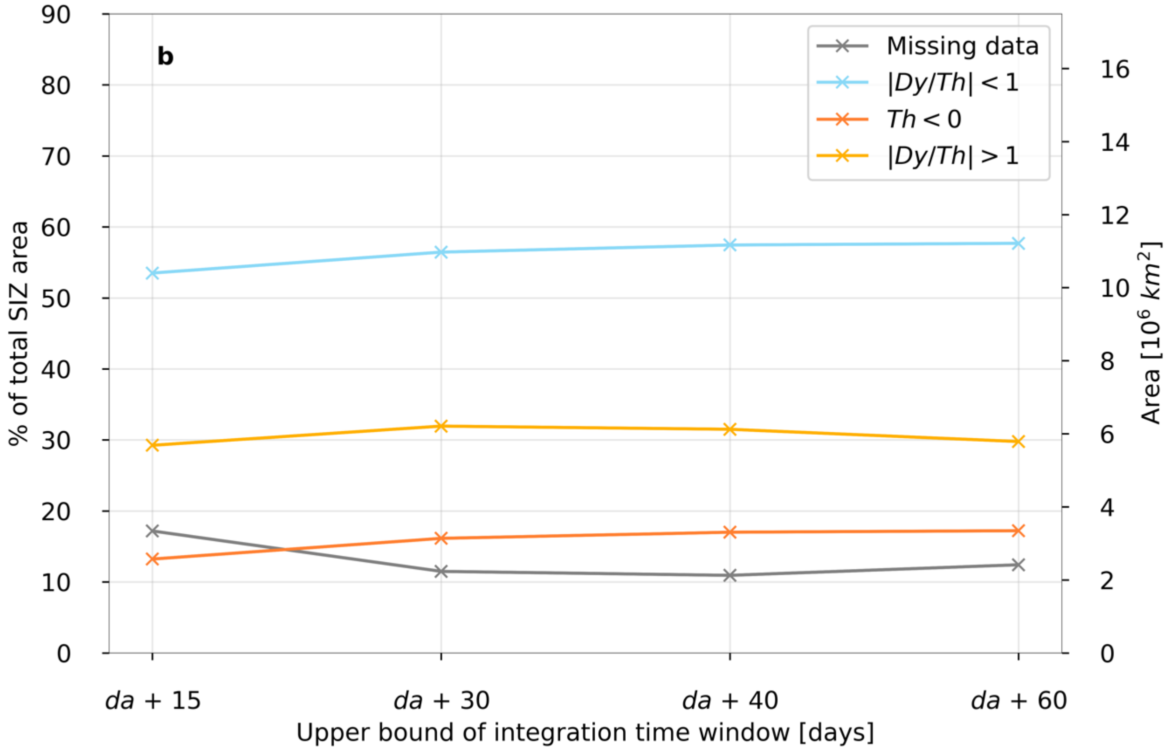

Supplementary Figure 1 | Sensitivity of the sea ice concentration-budget-based regions to the time-integration window. a shows the spatial distribution of three regions defined based on the budget: melting (Th < 0; orange), dominant dynamics (|Dy/Th| > 1; yellow and orange), dominant thermodynamics (|Dy/Th| < 1; blue). The budget is averaged after the date of sea ice advance (d_a), over a time window of unconstrained duration, which influence is tested here (values of 15, 30, 40 and 60 days). Grid points where the budget is not defined because of missing ice drift data are in grey and the perennial ice zone in white. **b** shows the fractional area of the aforementioned regions (in % of the seasonal ice zone), for the different integration time windows.

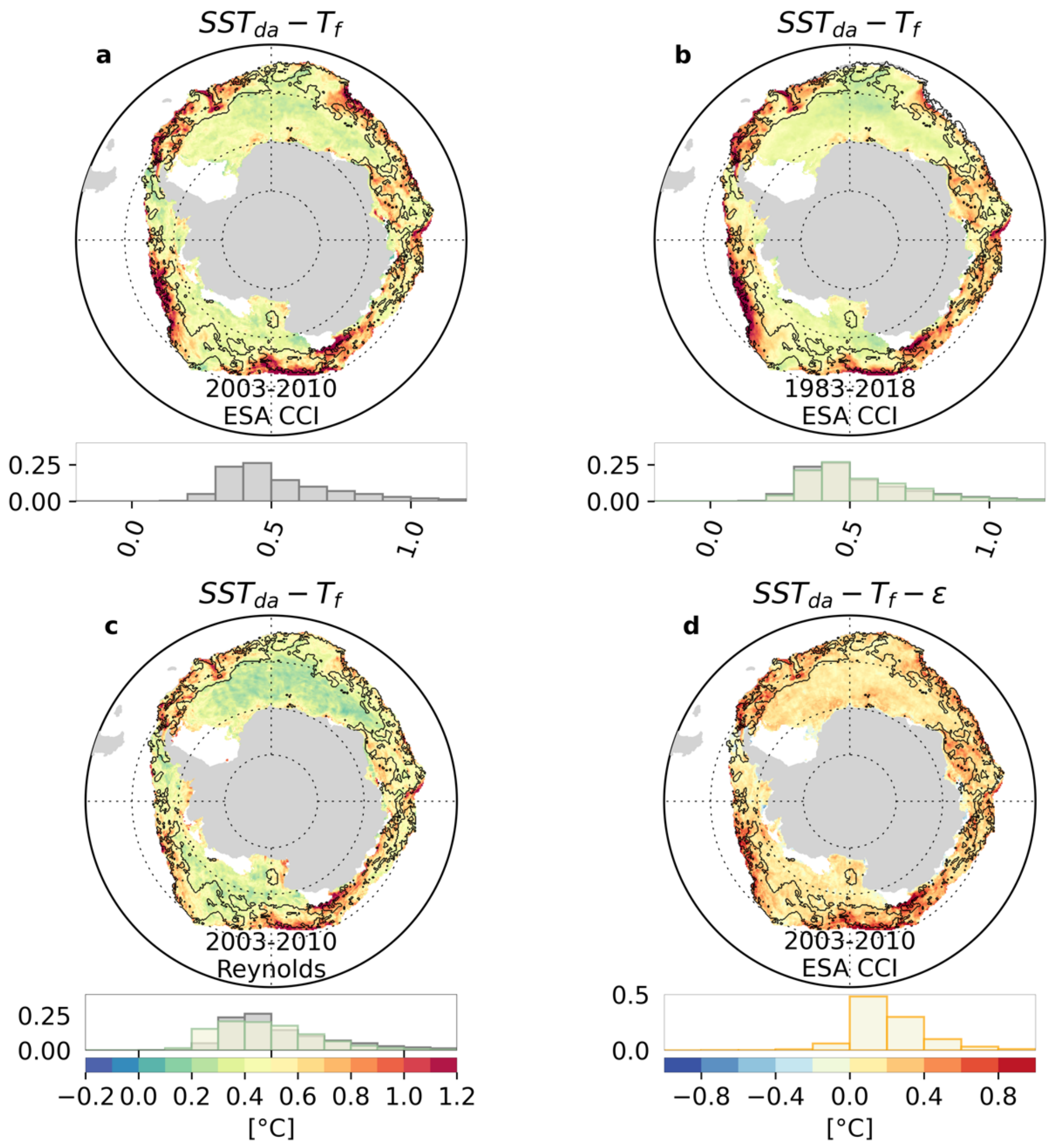

Supplementary Figure 2 | Robustness of the inner-outer zone limit across time periods and sea surface temperature (SST) products. SST at the date of advance (SST_{da}) referenced to freezing temperature (T_f, assumed constant at -1.8°C), based on **a** ESA CCI SST, averaged over 2003-2010; **b** ESA CCI SST, averaged over 1982-2018; and **c** Reynolds' SST, averaged over 2003-2010. **d** Difference between SST_{da} and T_f and the uncertainty (ε) on the ESA CCI SST analysis, averaged over 2003-2010. Corresponding frequency histograms are shown under each map in grey (**a**) or beige (**b**, **c**, **d**). In **b** and **c**, the grey histogram is the same as in **a** and used as a reference distribution. The black contour defines the limit between the inner and outer zones. White patches indicate regions out of the seasonal ice zone.

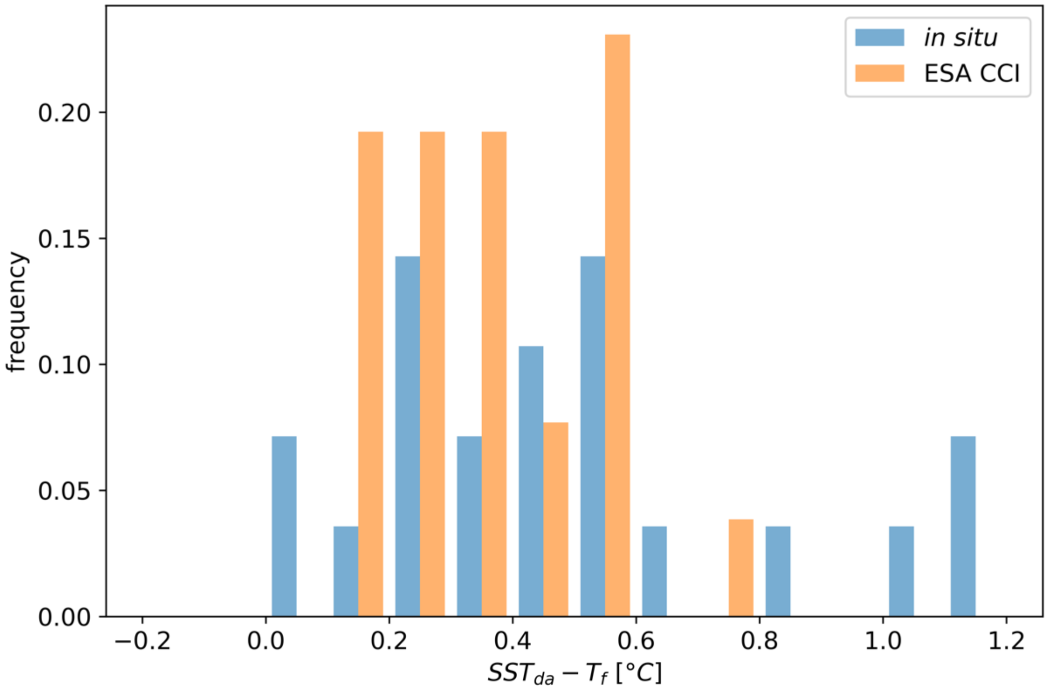

Supplementary Figure 3 | Comparison of satellite and in situ sea surface temperature on the day of advance (SST_{da}). Frequency histograms of SST at the date of advance referenced to freezing temperature (T_f, assumed constant at -1.8°C), derived from satellite (ESA CCI) and from in situ SST.

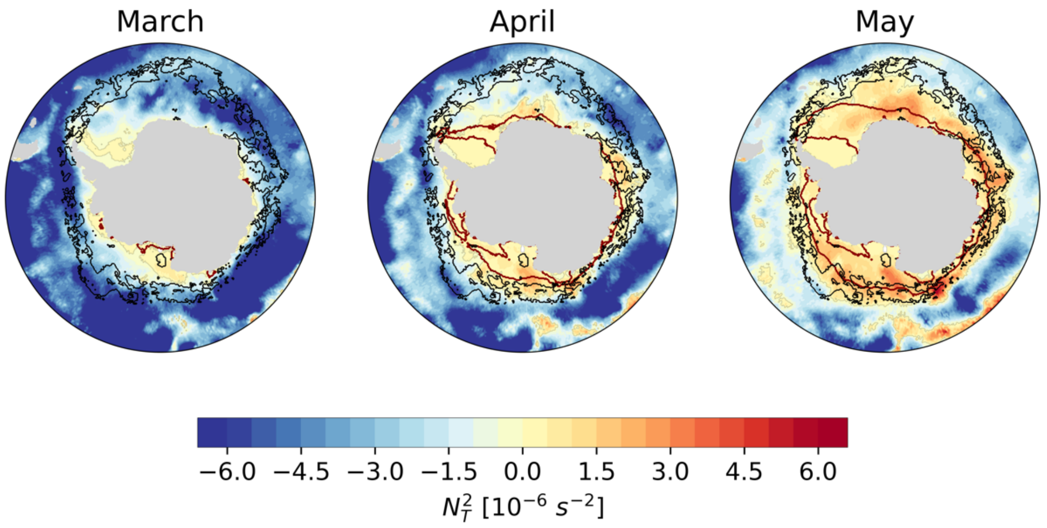

Supplementary Figure 4 | Monthly temperature contribution to the stratification at the base of the mixed layer (N_T^2) during the sea ice advance season. Maps are climatological, and constructed from in situ observations over 1979-2018. Red contours define the monthly climatology (over 1982-2018) of sea ice extent of the corresponding month, derived from passive microwave data. The black contour defines the limit between the inner and outer zones.

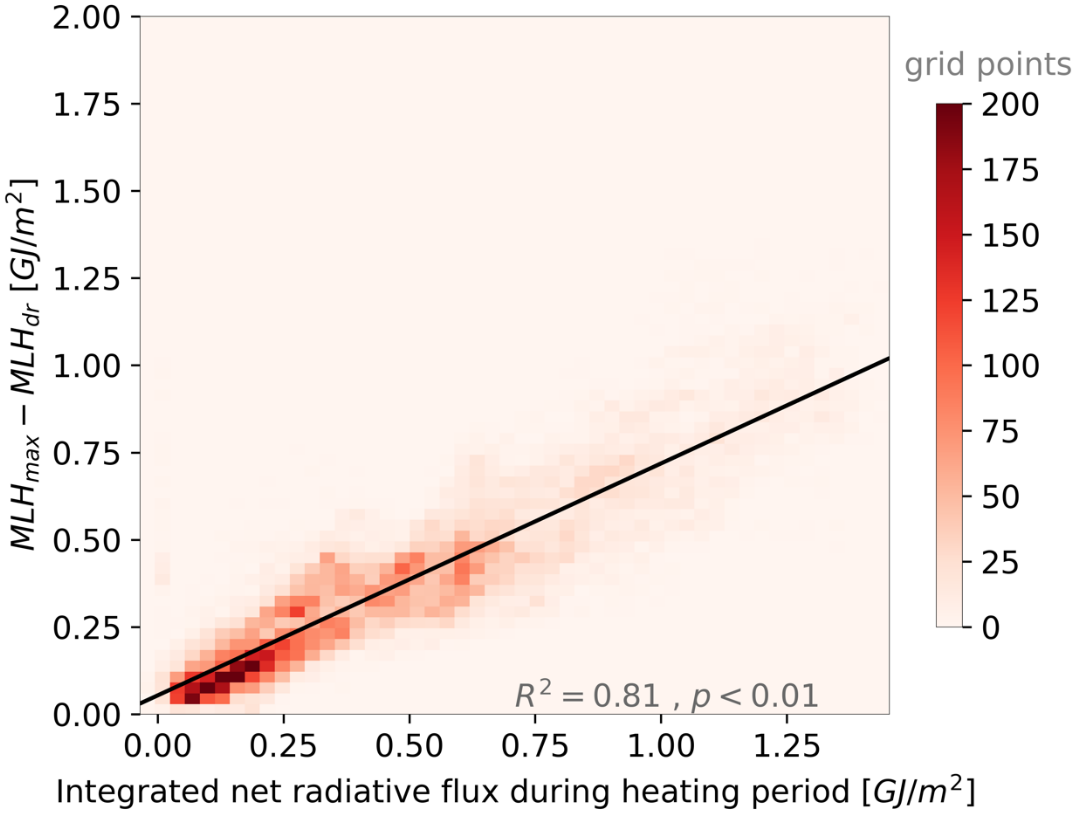

Supplementary Figure 5 | Spatial relationship between the increase in the mixed-layer heat content (MLH) and the total surface radiative heating over the ocean heating period (from the date of retreat to the date of seasonal maximum MLH). The sources used include: the climatological MLH derived from thermal infrared radiance satellite sea surface temperature (ESA CCI) and a climatology of mixed layer depths (1979-2018); the ISCCP observed climatological net radiative flux (1984-2016). The relationship is represented as a 2D histogram showing grid points from the inner zone only. Color gives the number of points in each pixel of the 2D histogram space. A Least Square linear regression was performed and the corresponding regression line, R^2 and p-values are shown.

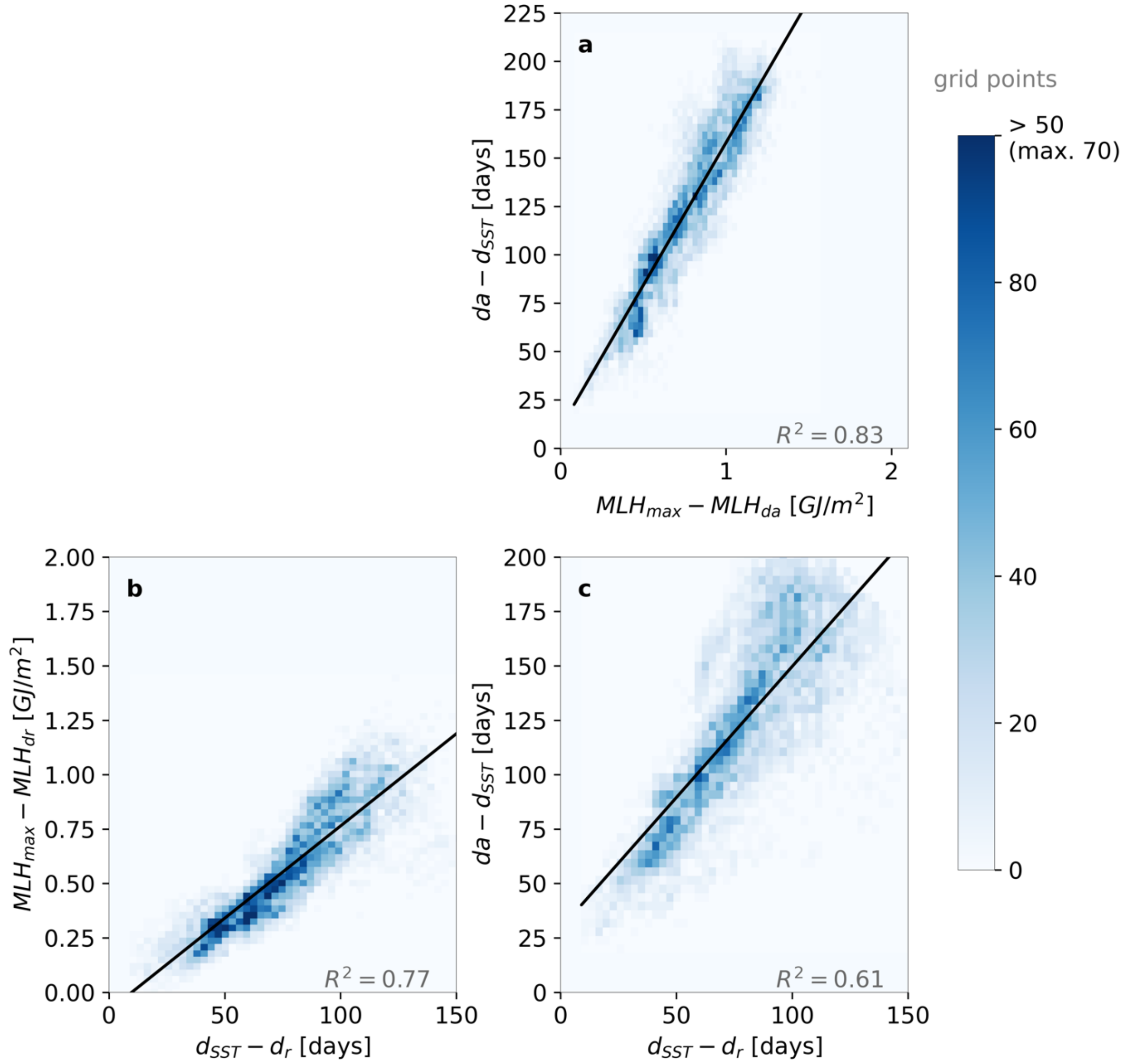

Supplementary Figure 6 | Selected outer zone spatial relationships between the 1982-2018 climatological maps of key variables displayed in Fig. 1, plotted as 2D histograms. a, date of advance (d_a) vs seasonal maximum of mixed layer heat content (MLH_{max}), **b**, MLH_{max} vs date of retreat (d_r) and **c**, d_a vs d_r. Anomalies are used, tailored to best showcase the relevant relationships (see Methods). d_a (d_r) anomalies refer to the date of maximum sea surface temperature (d_{SST}) such that positive anomalies indicate later advance (retreat). In **a** (**b**), MLH_{max} anomalies refer to the mixed layer heat content value at sea ice advance (retreat) date, which is close to but not exactly zero, because the sea surface temperature is a few tenths of degree above freezing (see Supplementary Figure 2). Only grid points from the outer zone are shown. Color gives the number of points in each pixel of the 2D histogram space. A Least Square linear regression was performed for each plot; the corresponding regression line (significant at 99%), and corresponding R^2 are given.

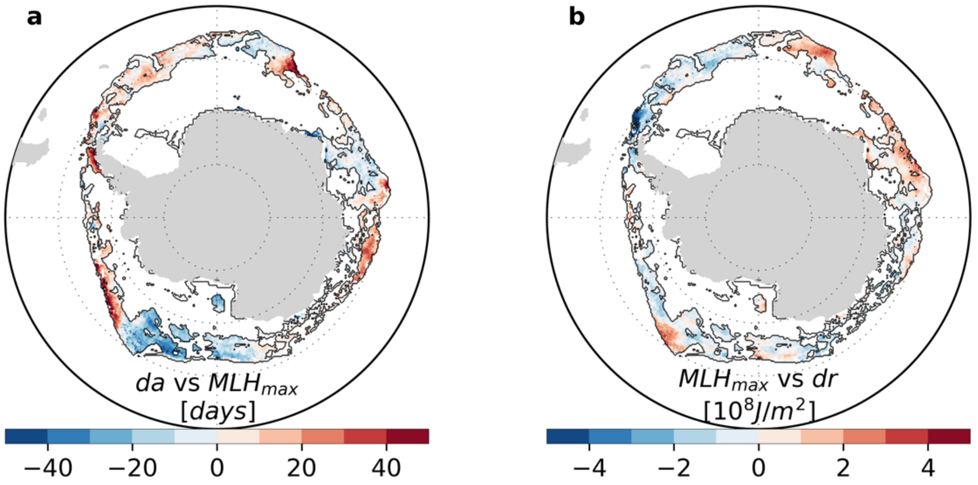

Supplementary Figure 7 | Spatial distribution of errors in the d_r-MLH$_{max}$-d_a linear regressions, in the outer zone. Error are defined as the residuals of **a**, the date of advance (d_a) vs seasonal maximum of sea surface maximum (MLH$_{max}$) and **b**, the MLH$_{max}$ vs date of retreat (d_r) linear regressions (Supplementary Figure 6). Residuals are calculated as the difference between the actual value at a given grid point and the predicted value based on the model fit. Only grid points located in the outer zone are displayed.
